# United States Department of Agriculture, Bureau of Animal Industry

**Published:** 1902-12

**Authors:** D. E. Salmon

**Affiliations:** Chief of Bureau


### SANITARY CONTROL WORK.

### UNITED STATES DEPARTMENT OF AGRICULTURE, BUREAU OF ANIMAL INDUSTRY.—CIRCULAR No. 38.

D. E. SALMON, D.V.M.,
CHIEF OF BUREAU.

### FOOT-AND-MOUTH DISEASE; WARNING TO ALL OWNERS OF CATTLE, SHEEP, AND SWINE.

### *Why this Circular is Issued.*

FOOT-AND-MOUTH disease of cattle, sheep, and other ruminants, and swine has recently been brought from some foreign country, and has appeared in a few localities in Massachusetts and some adjacent States. Since this disease has been unknown in America for many years, and then but to a limited extent, there are few who have practical knowledge of its nature. As it is vastly in the interest of all owners of cattle, sheep, and swine that this disease shall be eradicated promptly, and as they can render important aid, this circular of information is commended to their careful attention.

### *What Foot-and-Mouth Disease Is.*

This disease is an excessively contagious malady peculiar to ruminating animals (cattle, sheep, goats, deer) and swine. Rarely is it transmitted to man. It is characterized by the eruption of vesicles or blisters in the mouth, upon the heels, or between the toes, and upon the teats or udder. The appetite is depressed, the milk flow diminishes, the animal loses condition and becomes lame. After a day or two the vesicles break, peel off, and leave a raw surface that may heal in a few days, or, especially upon the feet and teats, that may remain sore for a long time and lead to serious complications. The death rate is very low, but it attacks the whole herd and many animals are seriously damaged, so that the loss to a herd owner is heavy.

### *The Importance of Exterminating this Outbreak Here and Now.*

European cattle owners have learned by long and bitter experience that this disease is the source of most discouraging, and not infrequently of ruinous losses. While the disease does not often kill, it damages, temporarily or permanently, every cow it attacks to the extent of from $10 to $40. The total loss on a herd is usually enough to wipe out a dairyman's profits for a year or two. The effect upon fat animals is quite as serious. It is not uncommon for the stock owners of England, France, or Germany to be injured by this disease, in a single year, to the extent of $5,000,000. With our much larger holdings of live-stock in this country, the possible losses from this disease, if it were to become general, are stupendous and incalculable. At present the disease exists over a comparatively small area. It is confidently believed by the experts who have investigated the situation that it can be controlled and eradicated. It is important that this shall be done, not only that the other parts of the country shall be protected, but also to prevent the frequent visitations of the disease that otherwise would afflict the live-stock of New England. To this end the aid of all stockmen and farmers is requested.

### *How Foot-and-Mouth Disease is Spread.*

There is no other disease that is so readily and certainly conveyed by contact. It is also conveyed by exposing healthy animals, even for an instant, to the stables, yards, pastures, or cars that have been occupied by affected animals; by buckets, cloths, brushes, or other objects that have been used by or on diseased cattle; by the use of forage exposed in mangers or even in the distant parts of the stable harboring infected animals. The disease is also carried by small animals, as dogs, cats, rats, birds, or upon the hands, boots, or clothing of men. A road along which diseased cattle have passed may retain enough virus to infect other cattle that pass over the same place several hours later. Premises occupied by diseased cattle are not safe for other cattle for a few months after the disease has disappeared. In short, it is to be remembered that every diseased animal is dangerous, and also every animal, person, or thing that has been near it or has been near a place occupied by it. Inspectors may avoid the danger of carrying the disease by cleanliness and disinfection.

### *How Foot-and-Mouth Disease May be Recognized.*

The symptoms of this disease most obvious to stockmen are: sluggishness, shivering, poor appetite, stiffness or lameness, collection of saliva upon the lips, slavering, slobbering or drooling, sucking and swallowing motions of the mouth and throat, smacking of the lips, blisters inside the lips, upon the gums, tongue, or roof of the mouth; later, raw sores in the same places. Blisters and sores may also form upon the teats or udder and upon the heels and between the toes. The flow of milk lessens or ceases, and the subject usually loses weight. All these symptoms may not be present in the same animal, and all are never present in an animal at one time. Moreover, the symptoms occur in varying degrees of severity. They may be very mild or very intense. The later symptoms may be intense lameness, emaciation, sore teats, and garget. With sheep and swine the feet are chiefly affected.

### *What Owners May do to Protect Their Stock Now and for the Future.*

The most important matter is to prevent the infection of animals not yet exposed. This can be done by avoiding the purchase of affected stock ; by excluding all outside animals from the herd or flock ; by each person who comes near healthy stock avoiding contact with diseased animals or the places or things contaminated by them ; by excluding visitors from the cow stable, sheep, and hog pens ; and by preventing the access of strange or stray animals, which may carry the virus on their feet or hair, although they are themselves in good health. Neither cows nor bulls should be removed from one place to another for service.

Should the herd or flock become infected, the appearance of the first evidence of disease should be immediately reported to the Chief of the Cattle Bureau, a State Cattle Commissioner, the local inspector of live animals, or to the Bureau of Animal Industry office, 147 Milk Street, Boston, Mass.

### *Urgent Necessity of Immediate Report of First Symptoms Causing Suspicion.*

The eradication of this disease and the removal of all quarantine and other restrictions upon the cattle trade can be materially hastened by the live-stock owners themselves, if they will promptly report the first evidence of foot-and-mouth disease in their herds or localities. This fact cannot be suppressed, and the sooner it is brought to the notice of the proper authorities the less the resulting damage will be. It is to be hoped that citizens everywhere will realize the importance of aiding the authorities who are working to eradicate this destructive plague, and they can render no more valuable service to themselves, their localities, or the nation than to immediately report a newly-infected animal or place.

*Fine for Neglect to Report the Disease.*

The law of Massachusetts imposes a fine of $100 on any person who suspects the existence of this disease and fails to report it in writing to the Chief of the Cattle Bureau, or his authorized representative.

D. E. SALMON,
Chief of Bureau.

Approved:

JAMES WILSON,
Secretary of Agriculture.

WASHINGTON, D. C., December 4, 1902.

